# Hydroxypropyl Methylcellulose-Based Nasal Sprays Effectively Inhibit In Vitro SARS-CoV-2 Infection and Spread

**DOI:** 10.3390/v13122345

**Published:** 2021-11-23

**Authors:** Kirsten Bentley, Richard J. Stanton

**Affiliations:** Infection & Immunity, School of Medicine, Cardiff University, Heath Park, Cardiff CF14 4XN, UK; BentleyK@cardiff.ac.uk

**Keywords:** SARS-CoV-2, nasal spray, hydroxypropyl methylcellulose

## Abstract

The ongoing coronavirus disease (COVID-19) pandemic has required a variety of non-medical interventions to limit the transmission of the causative agent, severe acute respiratory syndrome coronavirus 2 (SARS-CoV-2). One such option is over-the-counter nasal sprays that aim to block virus entry and transmission within the nasal cavity. In this study, we assessed the ability of three hydroxypropyl methylcellulose (HPMC)-based powder nasal sprays, produced by Nasaleze, to inhibit SARS-CoV-2 infection and release in vitro. Upon application, the HPMC powder forms a gel-like matrix within the nasal cavity—a process we recapitulated in cell culture. We found that virus release from cells previously infected with SARS-CoV-2 was inhibited by the gel matrix product in a dose-dependent manner, with virus levels reduced by >99.99% over a 72 h period at a dose of 6.4 mg/3.5 cm^2^. We also show that the pre-treatment of cells with product inhibited SARS-CoV-2 infection, independent of the virus variant. The primary mechanism of action appears to be via the formation of a physical, passive barrier. However, the addition of wild garlic provided additional direct antiviral properties in some formulations. We conclude that HPMC-based nasal sprays may offer an additional component to strategies to limit the spread of respiratory viruses, including SARS-CoV-2.

## 1. Introduction

Severe acute respiratory syndrome coronavirus 2 (SARS-CoV-2) is a large, enveloped virus belonging to the *Coronaviridae* family and is the causative agent of the coronavirus disease (COVID-19) pandemic that has, to date, infected over 194 million people and caused more than 4 million deaths worldwide (https://covid19.who.int/ (accessed on 23 September 2021)). The transmission of respiratory pathogens, including SARS-CoV-2, from person to person occurs during activities such as sneezing, coughing, and talking, via the generation of aerosols that contain droplets of varying sizes [1,2,3,4]. Transmission can occur in one of two ways: (1) virus-loaded droplets exhaled from an infected person coming into contact with the eyes, nose, or mouth of an uninfected person; or (2) droplets that have settled on surfaces and are subsequently transferred to an uninfected person’s hands, who then touches their eyes, nose, or mouth. Current evidence suggests that airborne and droplet particle transmission via direct contact with the eyes, nose, or mouth is a likely form of transmission of SARS-CoV-2 [5,6,7]. This has formed the scientific basis for many of the non-pharmaceutical interventions in the fight against COVID-19, such as mask wearing and maintaining a social distance of at least 2 metres.

Over-the-counter nasal sprays offer an additional measure for both the prevention of infection, and the spread thereafter, of respiratory pathogens. While a number of products incorporate small drug molecules or reactive species—for example, reactive oxygen and nitric oxide [8]—that actively target the virus, many rely on the creation of a physical barrier capable of blocking virus uptake. These passive barriers are generated via semi-synthetic or natural gelling agents such as hydroxypropyl methylcellulose (HPMC) [9] or carrageenan [10]. Carrageenan has been well studied in vitro, with formulations demonstrating an ability to block SARS-CoV-2 [11,12,13,14] and influenza [14,15] infection. Furthermore, clinical studies have demonstrated the potential to reduce the disease burden, revealing the potential of nasal sprays in real-world scenarios [16]. HPMC has been less well studied but has recently shown efficacy against coronavirus 229E [17] and the ability to block allergens such as pollen [18].

To improve the evidence for HPMC-based products, we assessed three HPMC-based nasal sprays from Nasaleze International Limited for their ability to block both infection with SARS-CoV-2 and the release of virus from cells previously infected with SARS-CoV-2. We show that the virus infection and release from cells was independent of virus variant and inhibited in a dose-dependent manner, with the optimum dose of 6.4 mg/3.5 cm^2^ inhibiting virus release over a 72 h period.

## 2. Materials and Methods

### 2.1. Cells and Virus

VeroE6 cells expressing ACE2 and TMPRSS2 (Vero A/T) were maintained at 37 °C, 5% CO_2_ in Dulbecco’s Modified Eagle Medium (DMEM) supplemented with 10% heat-inactivated foetal bovine serum (FBS). All SARS-CoV-2 work was carried out in an approved Category 3 facility. SARS-CoV-2 stocks were grown in standard VeroE6 cells in the presence of 2% FBS. All assays were conducted using DMEM supplemented with 2% heat-inactivated FBS and 50 µg/mL gentamycin (2%-DMEM).

For plaque assays, Vero A/T cells were seeded at 1 × 10^5^/well in 12-well plates 18 h prior to use. Samples were 10-fold serially diluted in 2%-DMEM to give a dilution series from 10^−1^ to 10^−6^ and cells infected for 1 h at 37 °C, 5% CO_2_. Virus inoculum was removed and cells overlayed with a 1:1 mix of 2.4% Avicel^®^ and 2× MEM (20% 10× MEM; 2% L-glutamine; 4% FBS; 5.4% sodium bicarbonate (7.5% soln.)). Cells were incubated for 72 h at 37 °C, 5% CO_2_, at which point overlay was removed, cells were washed once with phosphate-buffered saline (PBS) and fixed with 1 mL/well methanol for at least 5 min. Following removal of methanol, cells were stained with 0.1% (*w/v*) crystal violet, and plaques were counted.

### 2.2. Products

Test products were provided blind as products A, B, and C from Nasaleze International Limited. Upon completion of the study, product details were provided to aid data analysis. Product A: Nasaleze Cold/Travel Original (93% HPMC, 2% peppermint powder and 5% European wild garlic powder). Product B: Nasaleze Cold/Travel Allicin (95% HPMC, 2% peppermint powder and 3% allicin powder). Product C: Nasaleze Allergy (98.5% HPMC and 1.5% peppermint powder).

### 2.3. Inhibition of Virus Release

Vero A/T cells were seeded at 5 × 10^4^/well in 24-well plates 18 h prior to use. Cells were infected with SARS-CoV-2 (England-2) at a multiplicity of infection (MOI) of 1 or 0.01 and incubated for 30 min on a platform rocker at 37 °C, 5% CO_2_. Virus inoculum was removed, and cells were washed twice with PBS and 150 µL/well of fresh 2% DMEM added. To each well, either 1 mg, 3 mg, or 6.4 mg of dry powder product was evenly distributed across the cell monolayer and incubated at room temperature for 15 min for gel matrix formation to occur. Triplicate replicates were carried for each test condition, and duplicate experiments were conducted. A no-product/plus virus control well was also established on each plate. Once the gel matrix had formed, 1.5 mL of 2% DMEM was added to each well and the cells were incubated at 37 °C, 5% CO_2_ for the duration of the experiment. At 24, 48, and 72 h post-infection (hpi), 100 µL of supernatant was removed from each well and replaced with 100 µL of fresh 2% DMEM. Samples were stored at −80 °C until virus quantification by plaque assay, as above.

### 2.4. Inhibition of Virus Infection

Vero A/T cells were seeded at 5 × 10^4^/well in 24-well plates 18 h prior to use. Three types of assay plates were established: (1) test plate of product plus virus, (2) no-virus control plate with product only, and (3) no-product control plate with virus only. For test and no-virus control plates, growth media were removed from cells, replaced with 150 µL of fresh 2% DMEM, and 6.4 mg of dry powder product was evenly distributed within each well. Five replicate wells were established for each product tested. Plates were incubated at room temperature for 15 min for gel matrix formation. Once the gel matrix had formed, 1.5 mL of 2% DMEM containing SARS-CoV-2 (England-2, Alpha or Beta variant) at MOI 1 was added to each well. For the no-product control plate, 1.5 mL of 2% DMEM containing virus, as above, was added to each of the five replicate wells per virus strain. Additionally, five wells were established containing 1.5 mL of media only to act as an uninfected control. Cells were incubated at 37 °C, 5% CO_2_ for the duration of the experiment.

At 24, 48, and 72 h, plates were assayed for cell monolayer survival as a measure of the level of virus infection. Supernatant was removed from all wells, 1 mL/well of methanol added, and cells were incubated at room temperature for 1 h to fix. Methanol was removed and 0.5 mL/well of 0.1% crystal violet stain was added for 20 min. Crystal violet was removed, and cells were washed repeatedly with water until all of the product was removed from the cell monolayer. Crystal violet was added for a second incubation of 5 min to ensure all cells were evenly stained following the removal of the product. Crystal violet was again removed, the cells were washed once more with water, and the plates were left to dry.

Monolayer survival was determined by the level of crystal violet staining observed. To each stained well, 200 µL of 10% acetic acid was added and incubated for 15 min on a platform rocker to re-solubilise crystal violet. The samples were diluted 1:20 in water and the OD 595 was determined. Monolayer staining was expressed as a percentage of uninfected controls.

### 2.5. Suspension Test

A total of 6.4 mg of dry powder product was resuspended in 100 µL of DMEM to generate the gel matrix. To this was added 100 µL of virus inoculum containing approximately 2 × 10^6^ plaque forming units (PFU) of the England-2 strain of SARS-CoV-2The gel/virus mix was incubated for varying time periods at room temperature, before the addition of 1 mL of DMEM to diffuse the gel matrix. Residual virus was quantified by plaque assay, as above.

### 2.6. SARS-CoV-2 Nucleocapsid Detection Assay

Vero A/T cells were prepared as in Section 2.3 and infected with SARS-CoV-2 (England-2) at an MOI of 5 for 30 min on a platform rocker at 37 °C, 5% CO_2_. Cells were subsequently treated with 6.4 mg of product, as in Section 2.3. At 24 and 48 hpi, supernatant was removed and cells were fixed via the addition of 0.5 mL/well 4% (*v/v*) formaldehyde for 30 min. Cells were washed twice with PBS and 250 µL/well of NP-40 was added for 15 min to permeabilise cells. Cells were washed once with PBS-Tween (PBST) and blocked in 3% milk for 1 h in the dark. Block was removed and cells were stained with 250 µL/well of anti-nucleocapsid (N) primary antibody (SARS-CoV-2 Nucleocapsid Protein (1C7) Monoclonal Antibody; bsm-441411M; Generon, Slough, UK) and diluted in 1% milk in PBST for 1 h in the dark. Cells were washed once with PBST and 250 µL/well of secondary antibody (Peroxidase AffiniPure Donkey Anti-Mouse IgG (H + L); Jackson ImmunoResearch Europe Ltd., Ely, UK) was added in 1% milk in PBST for 1 h in the dark. Cells were washed once with PBST and levels of N were determined by the OD at 492 nm following the addition of OPD substrate as per manufacturers’ instructions (OPD Substrate Tablets; ThermoFisher Scientific, Waltham, MA, USA).

### 2.7. Statistical Analysis

**Figure 1:**
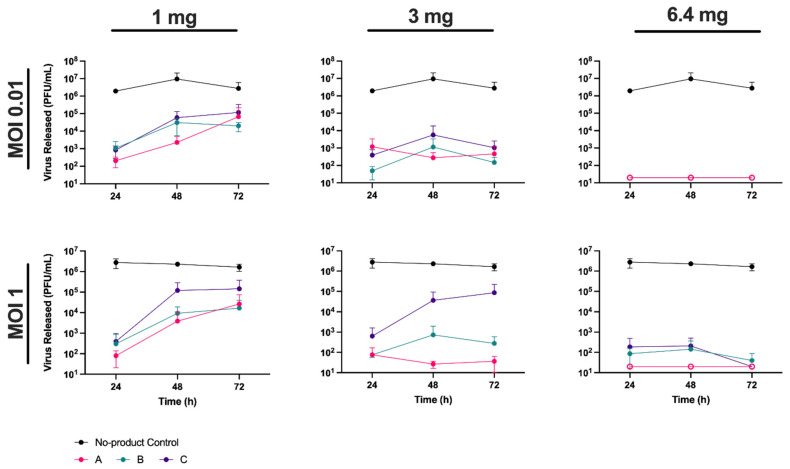
Hydroxypropyl methylcellulose inhibits severe acute respiratory syndrome coronavirus 2 (SARS-CoV-2) release in a dose-dependent manner. VeroE6 cells expressing ACE2 and TMPRSS2 (Vero A/T) in 24-well plates were infected with SARS-CoV-2 (England-2) at a multiplicity of infection (MOI) of 0.01 (upper panels) or 1 (lower panels) prior to addition of product A, B, or C, at 1 mg, 3 mg, or 6.4 mg per well. Supernatant samples were taken at 24, 48, and 72 h post-infection (hpi) and released virus quantified by plaque assay. Virus titres (plaque forming units (PFU)/mL) are shown for each product at each time point. Data points below the assay detection limit of 2 × 10^1^ PFU/mL are shown with open circles. Error bars represent the standard deviation of two experiments, each carried out in triplicate. See Table S1 for details of statistical analyses.

Statistical significance for all data was determined by two-way ANOVA, followed by Dunnett’s multiple comparisons test, and performed using GraphPad Prism version 9.2.0 for Windows, GraphPad Software, San Diego, CA, USA, www.graphpad.com. Data for Figure 1 and Figure 4 were log-transformed via GraphPad Prism prior to two-way ANOVA to obtain normal distribution of data points.

## 3. Results

### 3.1. Hydroxypropyl Methylcellulose Inhibits Release of SARS-CoV-2 in a Dose-Dependent Manner

To determine if HPMC could inhibit the release of virus from cells previously infected with SARS-CoV-2 we blind-tested three different powder nasal spray formulations from Nasaleze International Limited, referred throughout as products A, B, and C (see Materials and Methods for details). Vero A/T cells were first infected with the England-2 strain of SARS-CoV-2 at an MOI of 0.01 or 1 in 24-well plates. Following infection, cells were coated with each of the three test products to form a gel matrix that covered the cell monolayer (see Materials and Methods). Products were added at either 1 mg, 3 mg, or the manufacturers recommended dose of 6.4 mg per well, where one well represents a surface area of 3.5 cm^2^. The gel matrices were overlaid with 1.5 mL of media/well and incubated at 37 °C for 72 h. At 24, 48, and 72 hpi, 100 µL of supernatant was removed from each well and the amount of virus released was quantified by plaque assay (Figure 1).

We observed a clear dose response in the level of virus release. In the absence of product, virus concentrations reached 10^6^–10^7^ PFU/mL by 24 hpi, and were similar regardless of the MOI used in infection, highlighting the rapid growth of SARS-CoV-2 in cell culture. In the presence of products A and B, virus release was significantly (*p* < 0.01) reduced when compared to the no-product control at all time points. Product C also resulted in significant reductions in virus titre, although this reduction was notably less (*p* < 0.05) when present at 1 or 3 mg, and at later time points, in comparison to products A and B (Table S1). In contrast, in the presence of 6.4 mg of product, no significant difference in efficacy was observed between the three products. At this concentration, virus levels were generally below the detection limit of the assay (2 × 10^1^ PFU/mL), which corresponded to a >5-log (or >99.999%) reduction in virus release when compared to the no-product control (Figure 1). Thus, 6.4 mg of a powder nasal spray was sufficient to cover a surface area of 3.5 cm^2^ in a manner that effectively inhibited SARS-CoV-2 release from infected cells.

### 3.2. Hydroxypropyl Methylcellulose Blocks Infection with SARS-CoV-2

Having demonstrated that all three HPMC-containing products could effectively inhibit the release of virus from previously infected cells with a dose of 6.4 mg/3.5 cm^2^, we next investigated whether the nasal sprays could also inhibit de novo infection with SARS-CoV-2. To assess the inhibition of infection, formation of the product gel matrix was first required to cover the cell monolayer. Due to the nature of the gel matrix, its subsequent removal from cell monolayers was difficult without the removal process itself resulting in damage to the cell monolayer. As any damage to the monolayer would negatively bias the measurement of virus infection, we established a quantifiable assay that allowed cells to be fixed prior to determining the level of virus infection. SARS-CoV-2 infection of Vero A/T cells leads to destruction of the cell monolayer and thus could be used as a proxy measure for the level of infection by normalising monolayer survival—measured by residual crystal violet staining—of infected cells to uninfected cells. Additional controls were set up to measure monolayer survival in the absence of product, with or without SARS-CoV-2 infection (see Materials and Methods). Having established from the previous experiments that 6.4 mg was the most effective dose in a 24-well format, we proceeded with testing at this concentration, limiting infections to MOI 1. In addition, to determine whether virus strain had any impact on product effectiveness, we conducted infections with both the Alpha and Beta strains of SARS-CoV-2, as well as England-2, as tested above.

**Figure 2:**
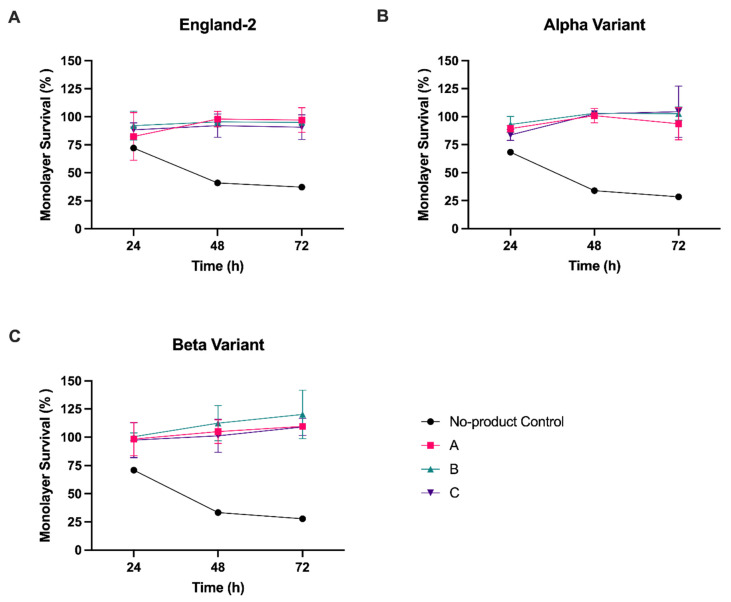
Hydroxypropyl methylcellulose inhibits SARS-CoV-2 infection. Vero A/T cells in 24-well plates were pre-treated with product A, B, or C, at 6.4 mg per well, followed by infection with (**A**) England-2, (**B**) Alpha, or (**C**) Beta strain of SARS-CoV-2 at MOI 1. Cell monolayer survival was determined at 24, 48, and 72 hpi and expressed as a percentage of uninfected control cells. Error bars represent the standard deviation of two experiments, each with five replicates. See Table S2 for details of statistical analyses.

In the no-product, virus-infected control cells, monolayer staining was reduced to ≤30% for all strains by 72 hpi (Figure 2 and Figure S1). In comparison, at 72 hpi we observed a significant (*p* < 0.001) increase in cell monolayer staining over the no-product control if virus infections were carried out on cells pre-coated with any one of the three nasal spray products (Figure 2, Table S2). The ability to block infection of the cell monolayer was also independent of the virus strain used for infection, with similar results obtained for all three SARS-CoV-2 strains tested.

### 3.3. Hydroxypropyl Methylcellulose Is Not Antiviral or Virucidal

As all three Nasaleze products had demonstrated an ability to inhibit both the amount of virus released from infected cells and de novo infection, we next sought to determine whether any of the products had direct antiviral or virucidal activity against SARS-CoV-2 that might be contributing to the inhibition observed.

**Figure 3:**
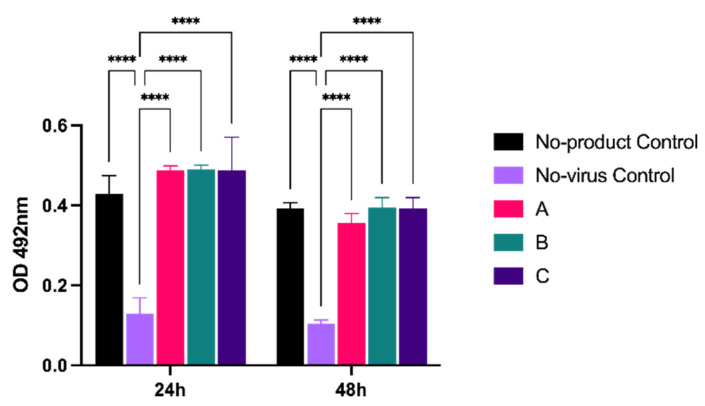
Virus replication is not reduced in the presence of product. Vero A/T cells in 24-well plates were infected with SARS-CoV-2 (England-2) at an MOI of 5 prior to the addition of product A, B, or C at 6.4 mg per well. At 24 and 48 hpi, cells were fixed and nucleocapsid protein levels were determined by antibody staining. Virus levels are expressed as the absorbance (OD) at 492 nm. Error bars represent the standard deviation of triplicate experiments. **** *p* ≤ 0.0001.

To determine whether products could directly inhibit virus replication, we adapted a microneutralization assay that we previously used to measure SARS-CoV2 replication in cells [19]. Vero A/T cells were infected at MOI 5, then overlaid with the highest concentration of product (6.4 mg), as per the previous experiment. Virus replication was assessed by antibody staining for the viral nucleocapsid (N) protein, a structural component that is required for virion assembly (Figure 3). In the presence of products A, B, and C, levels of N were comparable to a no-product control at both 24 and 48 hpi, and all were significantly (*p* < 0.001) higher than the no-virus control. Thus, cellular virus replication was not overtly inhibited by the presence of product.

**Figure 4:**
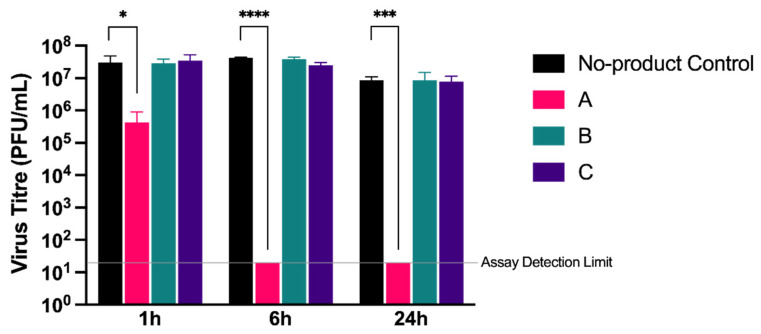
Determining virucidal activity of test products. The virucidal activity of products A, B, and C was determined by modified suspension assay. A gel matrix was formed with each product and subsequently incubated with England-2 strain of SARS-CoV-2 for 1, 6, or 24 h. The gel matrix/virus solution was diluted in DMEM and virus quantified by plaque assay. The amount of virus recovered is expressed as PFU/mL. Error bars represent the standard deviation of triplicate experiments. * *p* ≤ 0.05; *** *p* ≤ 0.001; **** *p* ≤ 0.0001.

Secondly, we assessed whether the products had direct virucidal activity against cell-free virions in a modified suspension assay. A total of 6.4 mg of each product was first resuspended in 100 µL of media to mimic the gel matrix created during the cell-based assays. A virus inoculum containing approximately 2 × 10^6^ PFU of SARS-CoV-2 (strain England-2) was added to the gel in a 1:1 (*v/v*) ratio and incubated at room temperature for 1, 6, or 24 h, after which the virus was recovered for quantification. No significant reduction in virus titre was observed for products B and C when compared to a media-only control at any time point (Figure 4). In comparison, product A showed a small but significant (*p* < 0.05) reduction in virus titre of 2-log_10_ at 1 h, with the virus subsequently undetectable following a 6 h incubation (*p* < 0.001). Unlike products B and C, product A also contained wild garlic powder (5%), supporting previous reports that garlic exerts potential antiviral activity [20].

These results suggest that, under the conditions tested, HPMC-based products are not inherently antiviral or virucidal, and that the mechanism of action is therefore via the formation of a passive barrier that physically blocks interaction of the virus at the cell surface. However, the inclusion of naturally derived antiviral products such as garlic may contribute to this physical blocking effect to reduce virus titres even further.

## 4. Discussion

One of the primary drivers in the spread of respiratory pathogens is airborne transmission via aerosols generated from infected individuals. The importance of preventative measures to block such transmission routes has been highlighted during the ongoing COVID-19 pandemic. While vaccination programmes against SARS-CoV-2 are proving highly successful at preventing severe disease, they do not completely stop infection and transmission, and worldwide vaccine uptake is highly variable due to a number of socio-political and economic factors. For this reason, additional preventative measures are likely to be required for some time. One such option is the use of nasal sprays that prevent the uptake and release of the virus. By functioning as a physical barrier, these products function irrespective of mutations that alter viral transmissibility, and do not need to be reformulated for different virus variants or strains; as a result, they may also form part of infection control measures in future viral pandemics, prior to the generation of specific vaccines.

In this study, we blind-tested three over-the-counter nasal sprays based on a powder form of HPMC for their ability to block the infection and spread of SARS-CoV-2 in an in vitro cell culture system. We observed a dose-dependent response in the ability of all products tested to inhibit virus release from cells previously infected with SARS-CoV-2, which was not a consequence of the inhibition of active virus replication. This is in agreement with studies on carrageenan-based nasal sprays that suggest these products exert their antiviral properties by inhibiting virus interactions at the cell surface [14,21]. Similarly, over-the-counter eyedrops have also been shown to act externally to genome replication [22], highlighting the more general mechanisms by which many of these non-pharmaceutical products act.

Although all products were similarly able to block the de novo infection of cells with SARS-CoV-2 at the highest dose tested, at lower doses there were some indications that product A was able to inhibit virus release to a greater degree than products B and C. For example, following infection at MOI 1, and in the presence of 3 mg of product, <10^2^ PFU/mL of virus was released by 72 hpi from product A compared to approximately 10^5^ PFU/mL from product C. This suggested that additional components in the nasal spray may be contributing to antiviral activity. In support of this, when tested against cell-free virions, product A reduced the viable virus to undetectable levels following a 6 h incubation, while products B and C showed no virucidal activity. In addition to peppermint powder—added for sensory purposes to all products—product A contained 5% wild garlic powder, while product B contained 3% allicin, an organosulphur compound extracted from garlic. Natural products, including garlic, have been increasingly investigated for their antibacterial [23] and antiviral [24] properties with a number of recent studies highlighting their potential against SARS-CoV-2 [25,26,27,28,29]. Given that the primary difference between product A and products B and C is the presence of wild garlic powder, it seems likely that this component is responsible for the virucidal activity. Interestingly, product B, which contains an extract of garlic in the form of allicin, did not show the same virucidal activity. The exact mechanism by which wild garlic powder exerts antiviral effects will therefore require further investigation. Nevertheless, it is clear that at higher concentrations, the formation of a physical barrier alone is sufficient to inhibit virus entry and release, while the addition of wild garlic has the potential to enhance this effect at lower concentrations.

Our study demonstrates high efficacy in an in vitro cell culture system; however, there are additional challenges in the use of nasal sprays in vivo. The nasal cavity is composed of a dual chamber measuring 5 cm high and 10 cm long, with a resulting total surface area of around 150 cm^2^ for both cavities [30]. When used as instructed, Nasaleze products deliver a dose of 6.4 mg to each nostril, with product concentrated at the start of the respiratory section of the nasal cavity, and as such would not be expected to cover the entirety of the nasal cavity surface area. In addition, the natural process of mucosal clearance in the nose results in the removal of product over time. Mucosal transport occurs at an average of 6 mm/min [30]. We can therefore estimate that it would take approximately 17 min for product applied within the main nasal cavity to traverse the 10 cm length of the chamber and be completely cleared, although in reality product clearance is unlikely to occur in such a consistent manner. Finally, virus can potentially infect and be secreted from both the mouth and nose, as well as in droplets originating in the lungs; therefore, not all sources of virus excretion would be inhibited by a nasal spray.

Despite these caveats, the nose remains the primary route by which air is inhaled, making the nasal cavity the predominant environment in which pathogens and other foreign particles are first targeted for removal, and a likely site for initial infection [31]. Limiting pathogen uptake via the nasal cavity therefore represents a valid target for the inhibition of viruses such as SARS-CoV-2 [11]. In support of this, clinical studies have demonstrated that carrageenan-based nasal barrier products reduce the duration of symptoms from coronavirus, influenza, and rhinovirus [16]. Our data suggest that HPMC-based nasal sprays may similarly play a role in limiting the spread and infection of SARS-CoV-2 via the nasal route, but application and dosage will be key to successful use. Our results also support the development of clinical studies to evaluate the real-world efficacy of HPMC-based products.

## Data Availability

All data are presented in the manuscript, and are available from the authors.

## Supplementary Materials

The following are available online at https://www.mdpi.com/article/10.3390/v13122345/s1, Figure S1: Plate images of inhibition of infection. Table S1: Statistical analysis for Figure 1. Table S2: Statistical analysis for Figure 2.
