# Supplementary material for: Hydroxypropyl Methylcellulose-Based Nasal Sprays Effectively Inhibit In Vitro SARS-CoV-2 Infection and Spread
Source: Viruses. 2021 Nov 23;13(12):2345. doi: 10.3390/v13122345 (PMC8705245; doi:10.3390/v13122345)
Supplement: Supplementary file 1 [file viruses-13-02345-s001.zip › viruses-1421971-supplementary.pdf]

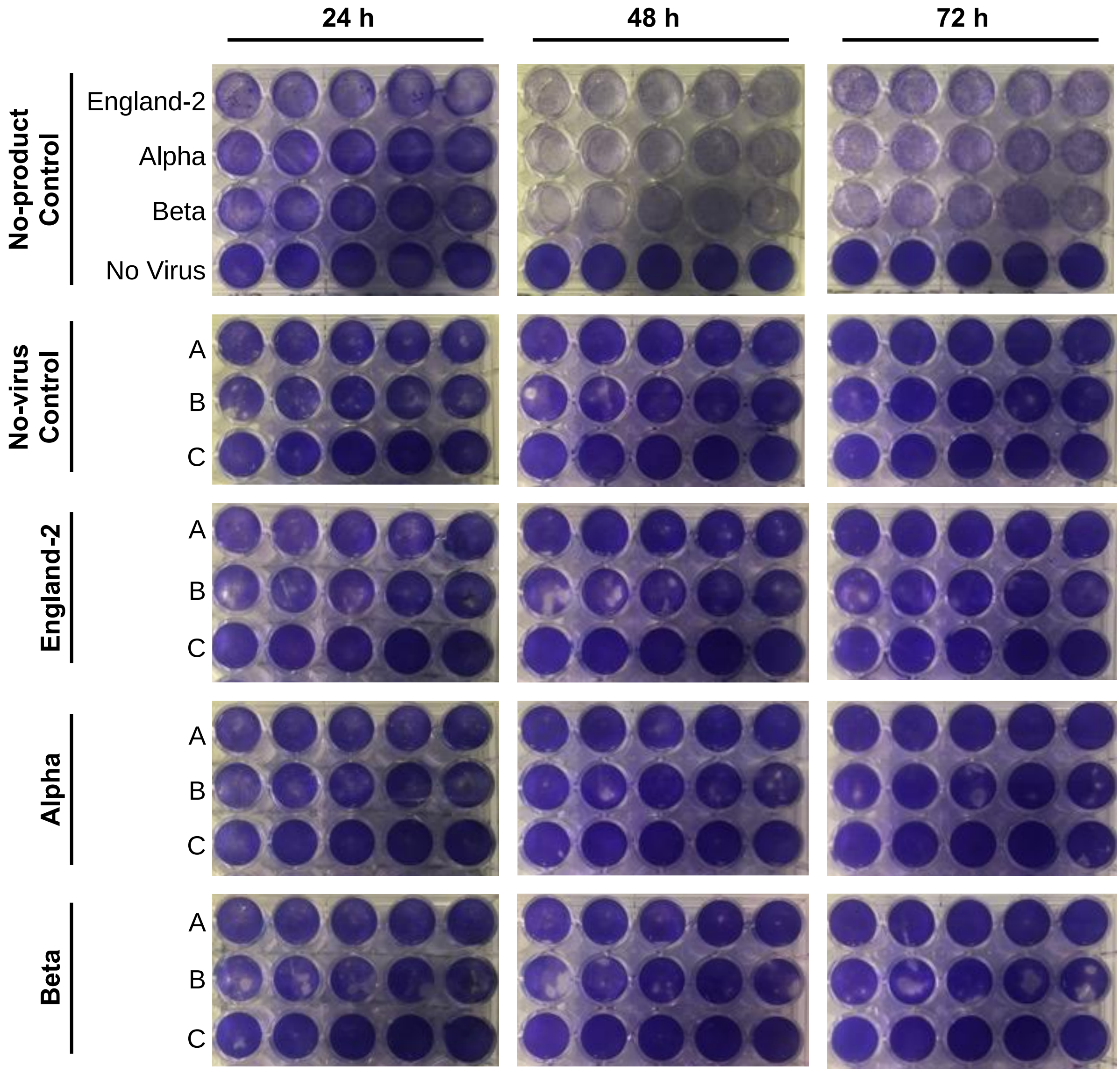

Figure S1. Plate images of inhibition of infection. Cell monolayer survival was used as a proxy measure for infection. Following pre-treatment with product A, B, or C, and SARS-CoV-2 infection of Vero A/T cells in 24-well plates, cells were fixed with methanol and stained with crystal violet. The plate images show the degree of monolayer staining, under each of the conditions tested, from one representative experiment.

Table S1: Statistical analysis for Figure 1

Test	MOI	Product	Timepoint	*P* value	Significance
**No-product *vs* A**	0.01	1g	24h	<0.0001	****
			48h	<0.0001	****
			72h	0.0052	**
		3g	24h	0.0003	***
			48h	<0.0001	****
			72h	<0.0001	****
		6.4g	24h	<0.0001	****
			48h	0.0004	***
			72h	0.0005	***
	1	1g	24h	<0.0001	****
			48h	0.0007	***
			72h	0.0074	**
		3g	24h	<0.0001	****
			48h	<0.0001	****
			72h	<0.0001	****
		6.4g	24h	0.0002	***
			48h	<0.0001	****
			72h	<0.0001	****
**No-product *vs* B**	0.01	1g	24h	<0.0001	****
			48h	0.0012	**
			72h	0.0011	**
		3g	24h	<0.0001	****
			48h	<0.0001	****
			72h	<0.0001	****
		6.4g	24h	<0.0001	****
			48h	0.0004	***
			72h	0.0005	***
	1	1g	24h	<0.0001	****
			48h	0.0005	***
			72h	<0.0001	****
		3g	24h	<0.0001	****
			48h	0.0002	***
			72h	<0.0001	****
		6.4g	24h	<0.0001	****
			48h	<0.0001	****
			72h	<0.0001	****
**No-product *vs* C**	0.01	1g	24h	<0.0001	****
			48h	0.0022	**
			72h	0.0069	**
		3g	24h	<0.0001	****
			48h	<0.0001	****
			72h	<0.0001	****
		6.4g	24h	<0.0001	****
			48h	0.0004	***
			72h	0.0005	***
	1	1g	24h	<0.0001	****
			48h	0.0033	**
			72h	0.0052	**
		3g	24h	<0.0001	****
			48h	0.0101	*
			72h	0.0173	*
		6.4g	24h	<0.0001	****
			48h	<0.0001	****
			72h	<0.0001	****

Table S2: Statistical analysis for Figure 2

Virus Strain	Product	Timepoint	*P* value	Significance
**England-2**	**A**	24h	0.4119	ns
		48h	<0.0001	****
		72h	<0.0001	****
	**B**	24h	0.0415	*
		48h	<0.0001	****
		72h	<0.0001	****
	**C**	24h	0.1145	ns
		48h	<0.0001	****
		72h	<0.0001	****
**Alpha**	**A**	24h	0.0804	ns
		48h	<0.0001	****
		72h	<0.0001	****
	**B**	24h	0.0368	*
		48h	<0.0001	****
		72h	<0.0001	****
	**C**	24h	0.2315	ns
		48h	<0.0001	****
		72h	<0.0001	****
**Beta**	**A**	24h	0.0830	ns
		48h	0.0001	***
		72h	<0.0001	****
	**B**	24h	0.0600	ns
		48h	<0.0001	****
		72h	<0.0001	****
	**C**	24h	0.0944	ns
		48h	0.0002	***
		72h	<0.0001	****
